# Spontaneous Rupture of the Testicle Secondary to Coughing in a Patient With New-Onset Heart Failure: A Case Report

**DOI:** 10.7759/cureus.81760

**Published:** 2025-04-05

**Authors:** Steven J Laxton, Jackie Dovgalyuk

**Affiliations:** 1 Emergency Medicine, University of Tennessee Health Science Center (UTHSC) Nashville - St. Thomas Health, Murfreesboro, USA

**Keywords:** cough, hemorrhagic shock, spontaneous testicular rupture, testicular rupture, urology

## Abstract

Spontaneous testicular rupture is an incredibly rare occurrence. There are no prior reported cases secondary to coughing or heart failure in the review of the current medical literature. There are other reports of testicular rupture, but these are secondary to trauma, malignancy, or infection, which are also rarely reported and otherwise not spontaneous. This is a report of a 68-year-old male who presented to the emergency department for complaints of pain and swelling in the scrotum and right groin that occurred after an episode of repeated coughing. The patient was found to have a spontaneous testicular rupture, causing an expanding hematoma that required operative management. The testicle, however, was not amenable to repair and required an orchiectomy to resolve the hemorrhage. The patient also presented with multiple additional medical problems requiring evaluation and medical optimization while hospitalized. Those additional problems were new-onset heart failure with reduced ejection fraction, cor pulmonale, and non-ST-segment elevation myocardial infarction (NSTEMI). The patient was ultimately able to be discharged home without complication from the spontaneous testicular rupture.

## Introduction

Testicular rupture is a rare phenomenon that occurs mostly in the setting of trauma (i.e., motorcycle or straddle accidents being the most common cause). There are other reports of testicle rupture occurring in relation to infection [1] and malignancy [2], but this is also rarely reported. The only reported rate of testicular rupture is 2.3 events per 1,000 trauma patients [3]; however, this is in the setting of trauma. For atraumatic testicular rupture, there is no reported incidence rate. 

In the review of the current literature, including PubMed and the National Institutes of Health database, there is currently no prior report of spontaneous testicular rupture secondary to coughing or heart failure as of March 2025. 

Currently, what is known concerning testicular rupture is that it is an uncommon injury that is characterized by a rupture of the tunica albuginea [4], the fibrous layer surrounding the testicle, resulting in exposure of the seminiferous tubules. When this fascial layer of the testicle is violated, it can cause the testicle to be compromised and be at risk of loss. Therefore, when suspected, prompt evaluation of these patients is crucial as a delay in surgical care may lead to loss or worsened injury of the testicle. 

In this case, the testicle was not viable and required a unilateral orchiectomy. Pathology examination of the testicle did not identify an underlying condition that would explain the etiology of the rupture, but intraoperative exploration showed bleeding from the testicular parenchyma that resolved with clamping the contents of the spermatic cord. Ultimately, the testicle was not amenable to repair and was excised.

## Case presentation

A 68-year-old male presented to the emergency department with complaints of sudden-onset testicular and right groin pain, as well as swelling. He has a past medical history of chronic tobacco use and prior remote myocardial infarction, after which he was lost to follow-up and has not seen a physician for many years. This patient's symptoms began approximately three hours prior to arrival, during which the patient said he was “coughing vigorously” and suddenly experienced pain and swelling in the right groin and right hemi-scrotum. Since that time, he has had worsening swelling and pain in the right groin and a notable ecchymosis to the groin and scrotum. The patient also endorsed a three-month history of gradual worsening diffuse swelling of the lower extremities that has progressed to his abdomen, shortness of breath on exertion, orthopnea, and intermittent left-sided chest pain. This is the patient's first time seeing a doctor for these symptoms. 

Physical exam showed findings significant for generalized anasarca to the mid-abdomen. Respirations were non-labored, but there were bilateral crackles present at the lung bases, and the patient was hypoxic, requiring 4 L/minute supplemental oxygen via nasal cannula. The patient was also noted to have diffuse firm swelling and ecchymosis of the penis and scrotum worse on the right side and throughout the right inguinal canal/groin. The patient had phimosis and ecchymosis to the inner thighs bilaterally. A repeat physical exam was performed approximately one hour after the initial presentation and showed further expansion of the swelling and ecchymosis in the right inguinal canal and scrotum, concerning an expanding hematoma. 

Labs were performed, including a complete blood count (CBC), basic metabolic panel (BMP), b-type natriuretic peptide (BNP), and troponin. The CBC and BMP were normal on initial evaluation. Repeat hemoglobin drawn three hours after surgery dropped 3 g/dL to 10.3 g/dL. The patient also developed clinical signs of circulatory shock that required transfusion of one unit of packed red blood cells. The troponin and BNP were abnormal, with significant elevation of both at 572 pg/mL (0-32 pg/mL normal) and 2,551 pg/mL (10-99 pg/mL normal), respectively. See Table 1 for the complete lab findings and reference ranges. These findings, in correlation with the patient’s electrocardiogram, are consistent with a non-ST-segment elevation (NSTEMI) and concern for new-onset heart failure. The NSTEMI in this case is likely a type 2 NSTEMI (demand ischemia) secondary to heart failure exacerbation. The patient did not have a coronary angiogram during hospitalization to evaluate for coronary blockage. 

**Table 1:** Laboratory findings on the complete blood count (CBC), basic metabolic panel (BMP), troponin, and b-type natriuretic peptide (BNP)

Lab	Result (*if abnormal)	Reference range
Basic metabolic panel		
Sodium (Na)	138 mmol/L	136-145
Potassium (K)	4.6 mmol/L	3.5-5.1
Chloride (Cl)	98 mmol/L	98-107
Carbon dioxide (CO_2_)	27 mmol/L	22-29
Blood urea nitrogen (BUN)	22 mg/dL	7-26
Creatinine (Ct)	0.8 mg/dL	0.7-1.3
Glucose	102 mg/dL	70-105
Cardiac enzymes		
B-type natriuretic peptide (BNP)	2,551.9 pg/mL *	10-99
Troponin	572 pg/mL *	0-35 in males
Complete blood count		
White blood cell (WBC)	13.4 x10^3/mm3 *	4.8-10.8
Red blood cell (RBC)	3.82 x10^6/mm3 *	4.2-5.4
HgG	13.1 gm/dL repeat 10.3 *	14-18
Hematocrit (Hct)	39.50%	40-52
Mean corpuscular volume (MCV)	103.4 fl *	78-98
Mean corpuscular hemoglobin (MCH)	34.3 pg *	26-34
Mean corpuscular hemoglobin concentration (MCHC)	33.2 gm/dL	32-36
Red cell distribution width (RDW)	14%	11.5-14.5
Platelets	221 x10^3/mm3	130-400

**Figure 1:**
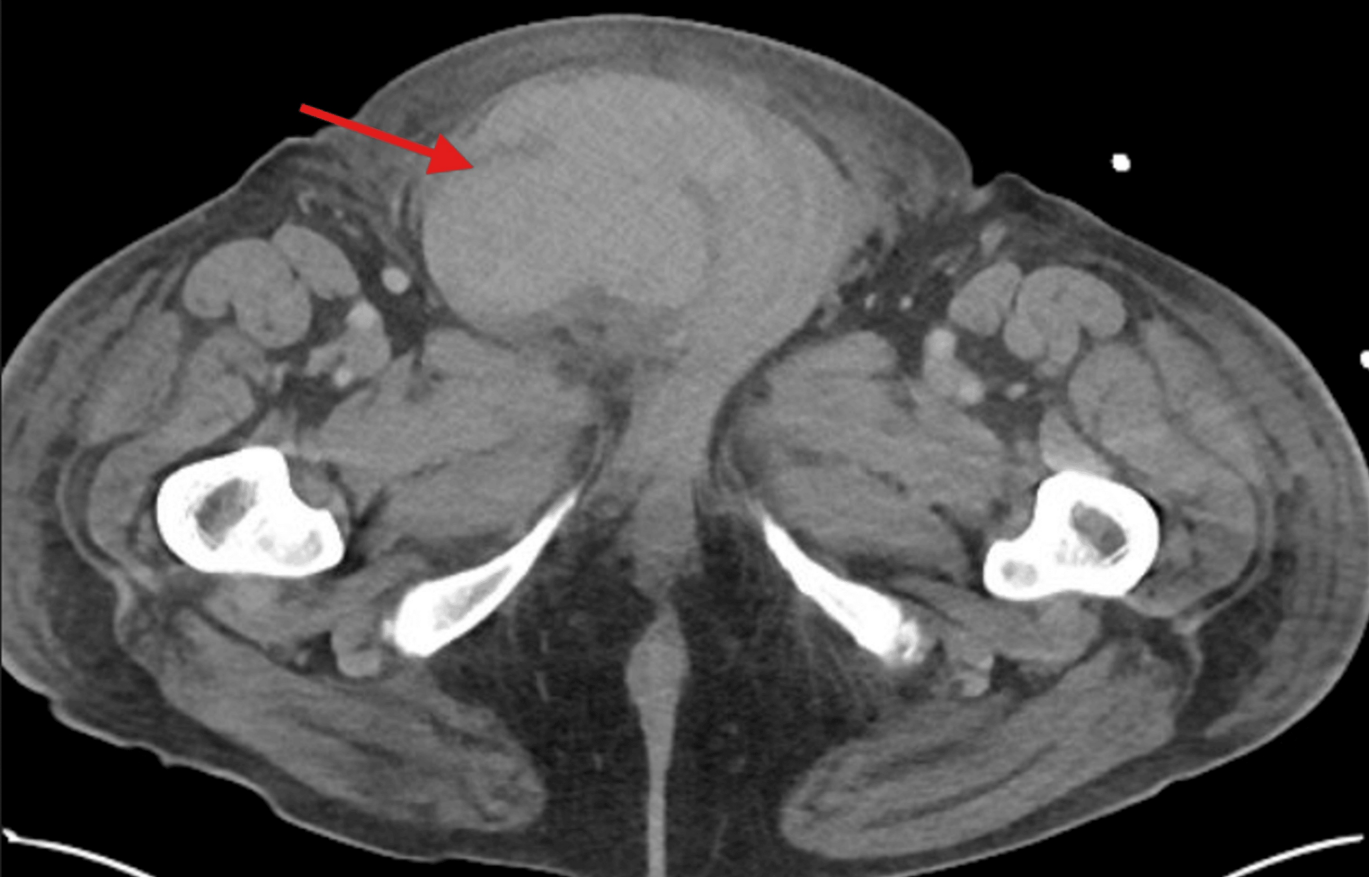
Hematoma formation within the distal right inguinal canal (red arrow)

**Figure 2:**
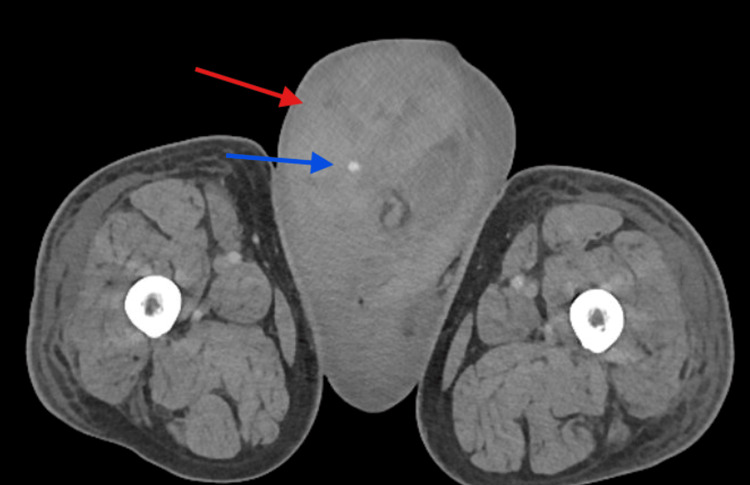
Expanding hematoma (red arrow) in the scrotum, worse in the right hemi-scrotum, with a puddling of contrast (blue arrow) suggestive of an expanding hematoma.

**Figure 3:**
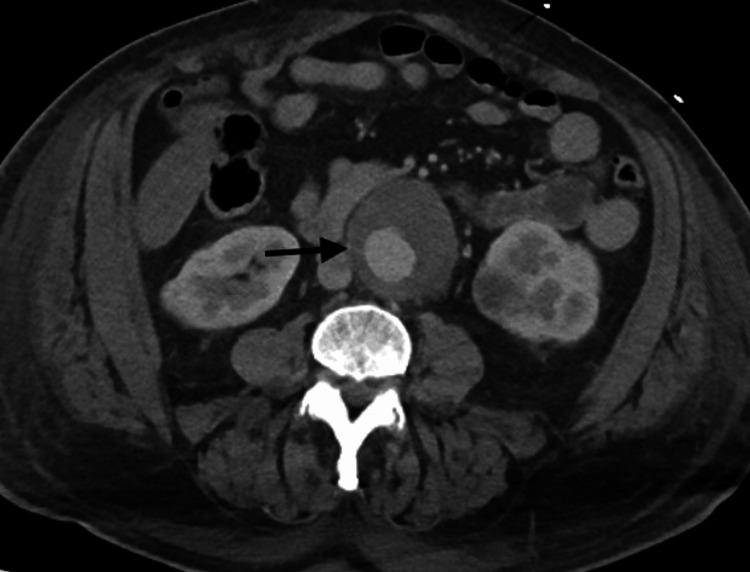
Fusiform infrarenal abdominal aortic aneurysm (black arrow)

A CT scan of the abdomen and pelvis with intravenous contrast showed a large hematoma in the right inguinal canal (Figure 1) extending into the scrotum. There was an area of hyperdensity thought to be a puddling of contrast, suggestive of possible ongoing arterial bleeding (Figure 2), which was consistent with the physical exam. There were also incidental findings of an infrarenal abdominal aortic aneurysm measuring 6.1 cm (Figure 3), as well as a solid mass at the inferior pole of the left kidney measuring up to 3.3 cm. Radiology indicated that the mass on the left kidney is likely reflective of renal cell carcinoma. 

CT scan of the chest with pulmonary artery angiography revealed no pulmonary embolus. However, it did show dense bibasilar consolidations and effusions likely reflective of volume overload, given the patient's physical exam findings of anasarca. 

Urology and General Surgery were both consulted while the patient was in the emergency department as it was initially unclear from where the expanding scrotal and groin hematoma originated. Interventional Radiology was also consulted, but they did not think they could safely access the source of the bleed. Urology ultimately took the patient to the operating room for exploratory surgery of the inguinal canal and scrotum, which revealed an expanding right scrotal hematoma, non-traumatic testicular rupture, and penile swelling with phimosis. 

In the operating room, a midline incision was made over the scrotal raphe and was carried down toward the right side. Almost immediately, a large hematoma was encountered. This was able to be dissected and evacuated, using primarily blunt dissection, away from the scrotal sac and testicle. At least 1 L of hematoma was evacuated. The right testicle was then encountered and was non-viable in appearance with evidence of testicular rupture. All of the planes in the scrotum were markedly abnormal due to the presence of hematoma. The testicular cord was also thickened with evidence of hematoma. The cord structures were then isolated. The cord was divided into four packets and clamped with hemostats. At this point, there was no active bleeding noted, and it was believed that the bleeding from the testicle was distal to the control of the cord. The testicle was excised and sent for pathology evaluation, which showed benign testicular parenchyma, epididymis, and spermatic cord. A foley catheter was successfully placed intraoperatively for penile phimosis. 

The patient had no further complications from the right orchiectomy and experienced routine wound healing. 

While hospitalized, the patient also underwent routine endovascular repair of his large abdominal aortic aneurysm that was without complication. 

The patient received further evaluation and medical optimization for NSTEMI and new-onset heart failure with reduced ejection fraction. An echocardiogram showed depressed ejection fraction of the left ventricle (28% measured by Simpson’s Biplane), findings of cor pulmonale with significant increase in size of the right heart chamber and moderate to severe reduction of systolic function, and akinesis of the right ventricle in the base that was consistent with McConnell’s sign. However, a CT chest with pulmonary artery angiography performed after the echo showed no pulmonary embolism, indicating that this finding was secondary to his left-sided heart failure. 

The patient was then discharged home after four days in intensive care for hemorrhagic shock and respiratory failure after the first surgery as well as seven days inpatient on the medical-surgical floor. 

## Discussion

The testicle is part of the male reproductive organ system that is paired and positioned within the scrotum. The blood supply normally emanates as a direct branch from the aorta just below the renal arteries and travels through along the abdominal wall, then exits the abdomen via the inguinal canal and travels into the scrotum and supplies the testicle. The blood supply then further divides into arterioles to supply the approximate 250 lobules and epididymis of the testicle. The testicle has a dense fascial covering called the tunica albuginea. 

Testicular rupture is defined as a fracture of the fascial coating of the testicle, called the tunica albuginea. Evaluation for this is best done by testicular ultrasound. The most specific findings on ultrasonography are loss of testicular contour and heterogeneous echotexture of parenchyma. The highest reported sensitivity for testicular rupture on ultrasound is 93% [5]. Testicular salvage rates are high and usually involve suture repair of the site of rupture. If unable to repair, then a simple orchiectomy should be performed [5]. 

In this case, the patient’s symptoms were preceded by “vigorous coughing,” which likely created changes in blood pressure, causing a repeated acute increase in arterial pressure [6] like what would be seen in phase one [7] hemodynamic change during a Valsalva maneuver. With his presentation of likely new-onset heart failure and echocardiography findings of severe cor pulmonale, it is proposed that the elevated venous pressure due to systemic venous congestion [8,9] and repeated spike in arterial blood pressure is what caused the right testicle to rupture spontaneously in this case. 

It is expected that spontaneous rupture of the testicle should not occur unless there is a weakened or already damaged point in the testicle to predispose this condition, like what is seen in other reports of spontaneous solid organ rupture [10,11] or prior reports where infection or malignancy in the testicle preceded rupture. However, in this case, the pathology report does not identify an underlying illness of the testicle such as malignancy, infection, granuloma, or vascular aneurysm formation that would predispose to this condition. There is no prior documented report of spontaneous testicular rupture due to heart failure or coughing. 

Another proposition of what occurred is aneurysmal rupture of the testicular artery at the insertion site or within the testicle parenchyma. As this patient was already found to have an abdominal aortic aneurysm, he is at an increased risk for aneurysm of additional arteries. There was no testicular artery aneurysm noted on the CT scan of the abdominal and pelvis, and no aneurysm was noted on operative exploration. There was also no noted aneurysm on the gross and microscopic pathology review of the testicle. This is also unlikely as prior case reports of testicular artery aneurysm occur at the origin of the artery near the aorta [12] or following trauma or infection [13]. There is one prior report of a pseudoaneurysm [14] within the spermatic cord, but once again, if this was present, this would have been recognized on gross and/or microscopic pathology review, making it unlikely. 

## Conclusions

Acute testicular rupture is a rare phenomenon mostly related to trauma, with prior reports related to testicular malignancy or infection. There are no prior reports of spontaneous testicular rupture without a preceding “defect” in the testicle, such as malignancy or infection.

Findings that would raise suspicion for this condition are testicular pain, scrotal bruising, and enlargement of the scrotum. If related to trauma, then suspicion should be much higher. If testicular rupture is suspected, urology should be consulted for definitive management, as operative exploration remains the gold standard for diagnosis and treatment of testicular rupture. 

In this case, the testicular rupture was preceded by coughing and findings of new-onset heart failure. It is suspected that this patient had a combination of systemic vascular congestion from heart failure, severe cor pulmonale, and repeated rise in systemic arterial pressure from repeated coughing that caused sudden rupture of the testicle.
